# Redoxomics in Ageing and Neurodegenerative Disorders: From Bench to Clinics

**DOI:** 10.37825/2239-9747.1045

**Published:** 2023-12-29

**Authors:** Vittorio Calabrese, Ursula M. Jacob, Tilman Fritsch, Ali S. Abdelhameed, Noemi Osakabe

**Affiliations:** a Department of Biomedical and Biotechnological Sciences, University of Catania, Italy; b System Biologie AG, Wollerau, CH, Switzerland; c NAM Institute, Salzburg, Austria; d Department of Pharmaceutical Chemistry, College of Pharmacy, King Saud University, Riyadh 11451, Kingdom of Saudi Arabia; e Department of Bio-science and Engineering, Faculty of System Science and Engineering, Shibaura Institute of Technology, Japan

*Dear Editor*,

Exploring molecular mechanisms of ageing and determinants of lifespan will help reduce age-related morbidity, thus facilitating healthy brain ageing. Recently, it has been demonstrated that nutritional polyphenols, the main constituents of the Mediterranean diet, maintain redox balance and neuroprotection through the activation of hormetic vitagene pathway. Mitochondria play pivotal roles in the mechanisms of cellular ageing and lifespan extension, although further studies are required concerning optimal bioenergetic mechanisms promoting aerobic energy production and the underlying detrimental effects of reactive oxygen species (ROS) by-production with the interplayed nutrition and caloric intake modulatory effects. Consistently, ROS acting as sensors of intracellular nutrients and energy state regulate functional mitochondrial state. Interestingly, increasing evidence reports a functional crosstalk between ROS production by mitochondria and longevity pathways modulating lifespan across species thus ensuring healthy ageing. Glutathione (GSH) is a tripeptide with multiple important functions in living organisms, including antioxidant defence and xenobiotic removal. γ-Glutamylcysteine (γ-GC) as an immediate precursor of glutathione (GSH) has been originally used for the treatment of sepsis, inflammation bowel disease, and senescence. γ-GC ligase is the rate limiting enzyme of Meister cycle, and hence central in the regulation of GSH biosynthesis and cellular redox state. Consistently, NF-E2-related factor 2 (NRF2) plays a crucial role in the maintenance of cellular homeostasis by regulating various enzymes and proteins that are involved in the redox reactions utilizing sulfur. While substantial impacts of NRF2 on mitochondrial activity have been described, the precise mechanism by which NRF2 regulates mitochondrial function is still not fully understood. Several studies reveal that nuclear factor erythroid 2-related factor 2 (Nrf2) regulates redox homeostasis and works as an anti-inflammatory in various degenerative disorders. Consistent to this notion, Nrf2-dependent pathways of cellular stress response with their target antioxidant *vitagenes* are emerging as powerful systems capable to preserve redox homeostasis under environmental and metabolic stresses. *Vitagenes* encode redox longevity genes induced by oxidative damage including heat shock family (Hsp) Hsp32, Hsp70, glutathione, thioredoxin and sirtuin protein systems. During aging process, a gradual decline of the heat shock response occurs and this may prevent repair of protein damage. Therefore, there is a growing interest by scientific community in developing of novel preventive and pharmacological agents capable of inducing optimized stress responses at the minimum dose within the broad framework of hormesis as a general therapeutic strategy in patients suffering from chronic degenerative diseases as well as a broad public approach to slowthe offset of ageing and age-related neurodegenerative processes. Relevant to inflammatory damage and its therapeutics, experimental models suggest that females defence against oxidative stress ismore proficient than males, determining a longer lifespan and lower incidence of most chronic diseases. Thus, interplay and coordination of redox interactions and their interaction with endogenous and exogenous antioxidant defence systems is an emerging area of reserach interest in anti-inflammatory anti-degenerative therapeutics, with special attention warranted to gender features of neurocognitive deficit, associated to functional and structural disorders occurring in ageing and neurodegenerative. Consistently, dietary polyphenols involved in the activation of *vitagenes* resulting in improved intracellular antioxidant defense systems against ROS damage leading to degeneration and death with considerable impact on brain health and longevity processes, may also present sex specific patterns of phenotypic expression [1–3].
